# Box–Behnken Design for Hydrogen Evolution from Sugar Industry Wastewater Using Solar-Driven Hybrid Catalysts

**DOI:** 10.1021/acsomega.2c05721

**Published:** 2022-11-08

**Authors:** Ceren Orak, Aslı Yüksel

**Affiliations:** † Department of Chemical Engineering, Izmir Institute of Technology, 35430 Urla, Izmir, Turkey; ‡ Geothermal Energy Research and Application Center, Izmir Institute of Technology, 35430 Urla, Izmir, Turkey

## Abstract

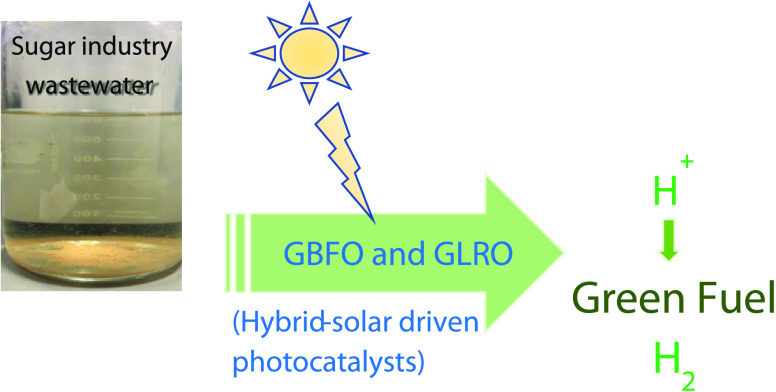

Hydrogen is a clean and green fuel and can be produced from renewable sources via photocatalysis. Solar-driven hybrid catalysts were synthesized and characterized (scanning electron microscopy (SEM), transmission electron microscopy (TEM), Brunauer–Emmett–Teller (BET), X-ray diffraction (XRD), photoluminescence (PL) spectroscopy, and UV–vis diffuse reflectance spectroscopy (DSR)), and the results implied that graphene-supported LaRuO_3_ is a more promising photocatalyst to produce hydrogen and was used to produce hydrogen from sugar industry wastewater. To investigate the main and interaction effects of reaction parameters (pH, catalyst amount, and [H_2_O_2_]_0_) on the evolved hydrogen amount, the Box–Behnken experimental design model was used. The highest hydrogen evolution obtained was 6773 μmol/g_cat_ from sugar industry wastewater at pH 3, 0.15 g/L GLRO, and 15 mM H_2_O_2_. Based on the Pareto chart for the evolved hydrogen amount using GLRO, among the main effects, the only effective parameter was the catalyst amount for the photocatalytic hydrogen evolution from sugar industry wastewater. In addition, the squares of pH and two-way interaction of pH and [H_2_O_2_]_0_ were also statistically efficient over the evolved hydrogen amount.

## Introduction

Hydrogen evolution from various renewable sources such as biomass and wastewater streams using solar energy to provide a simultaneous solution for current global energy and environment problems has recently gained great attention because it is considered to be a promising and green fuel in future. One of the most promising hydrogen evolution processes is photocatalysis since it involves green processes and also it is a simple and relatively low-cost process. Various semiconductors with different band gap energies were used to produce hydrogen from alcohol, glycerol, and wastewater.^1−4^ Kuang and Zhang synthesized carbon-doped TiO_2_ (C-TiO_2_) and reduced graphene-decorated C-TiO_2_ (C-TiO_2_/rGO) to produce hydrogen from water using methanol as an electron donor under visible light irradiation. The amount of hydrogen produced was 0.67 ± 0.12 to 1.50 ± 0.2 mmol g^–1^ h^–1^ using C-TiO_2_ and C-TiO_2_/rGO, respectively. Graphene has a large surface area and provides better electron transfer, thus enhancing the obtained hydrogen amount. Additionally, the band gap energies of C-TiO_2_ and C-TiO_2_/rGO were reported to be 2.5 and 2.2 eV, respectively. Hence, the introduction of rGO causes a decrease in the band gap energy, and so the utilization of visible light becomes a feasible option.^5^ Cheng et al. synthesized a hybrid catalyst (TiO_2_–graphene nanocomposite) via a solvothermal reaction to produce hydrogen from the mixture of methanol and water by photocatalytic oxidation, and the mass ratio of graphene was optimized to be 0.5 wt % and almost 6000 μmol of H_2_ was produced using this catalyst. Graphene served as an acceptor of the photogenerated electrons of TiO_2_ and as a transporter to separate the photogenerated electron–hole pairs effectively. The hybrid catalysts had enhanced light absorption ability and a lower recombination rate of photogenerated electron–hole pairs and hence showed higher photocatalytic activity toward hydrogen production from the methanol and water mixture under visible light illumination than raw TiO_2_.^6^ Sekar et al. studied hydrogen production from Bisphenol A, which is an endocrine-disrupting chemical and has severe toxic effects on human health, using a hierarchical bismuth vanadate (h-BiVO_4_)/reduced graphene oxide (rGO) composite. While 11.5 μmol g^–1^ h^–1^ of hydrogen was produced in the presence of h-BiVO_4_/rGO, 0.03 μmol g^–1^ h^–1^ of hydrogen was obtained using BiVO_4_. While 57% Bisphenol A removal was achieved in the presence of BiVO_4_, a 72% removal was obtained using h-BiVO_4_/rGO. Hence, the introduction of graphene causes an increase in the produced hydrogen amount and Bisphenol A removal since graphene has outstanding properties. For instance, it behaves as a cocatalyst, charge transfer mediator, photosensitizer, and electron trap.^7^ In addition, perovskite-type catalysts (i.e., LaCoO_3_, LaFeO_3_, CaTiO_3_) could be used to produce hydrogen by photocatalysis.^8−10^ For instance, Acharya et al. examined water decomposition reaction using the LaFeO_3_ nanotubes/graphene oxide (GO) composite via photocatalytic oxidation and using methanol as a sacrificial agent, and the maximum hydrogen production (611.3 mmol h^–1^ g^–1^) was obtained in the presence of LaFeO_3_ nanotubes/(1% by mass)GO. Furthermore, it has been found that the addition of reduced GO to LaFeO_3_ reduces band gap, and it was found that the reduced GO provided a more active surface for adsorption and photocatalytic reaction because of the wide surface area and that the reduced GO layer could efficaciously collect and transport electron, which reduced the probability of electron–hole recombination and increased the efficiency of separation.^11^ Therefore, considering the wide band gap range of perovskite-type catalysts (BiFeO_3_ and LaRuO_3_) and the outstanding properties of graphene, in this study, solar-driven hybrid catalysts, i.e., graphene-supported BiFeO_3_ (GBFO) and graphene-supported LaRuO_3_ (GLRO), were synthesized to produce hydrogen from sugar industry wastewater.

In the literature, various photocatalysts were tested to produce hydrogen from sucrose solution. For instance, noble-metal (Pd, Pt, Au, Rh, Ag, and Ru) loaded TiO_2_ and La-doped alkali tantalate were used.^12,13^ To the best of our knowledge, GBFO and GLRO have not been used to produce hydrogen from sucrose solution and/or sugar industry wastewater, and so in this study, hydrogen evolution from sucrose solution and sugar industry wastewater was performed using these solar-driven photocatalysts. Additionally, the Box–Behnken statistical model was used to investigate the impacts of reaction parameters (pH, catalyst amount, and [H_2_O_2_]_0_) on the evolved hydrogen amount, and their main impacts and interactions were statistically analyzed.

## Results and Discussion

### Characterization of Hybrid Catalysts

**Figure 1:**
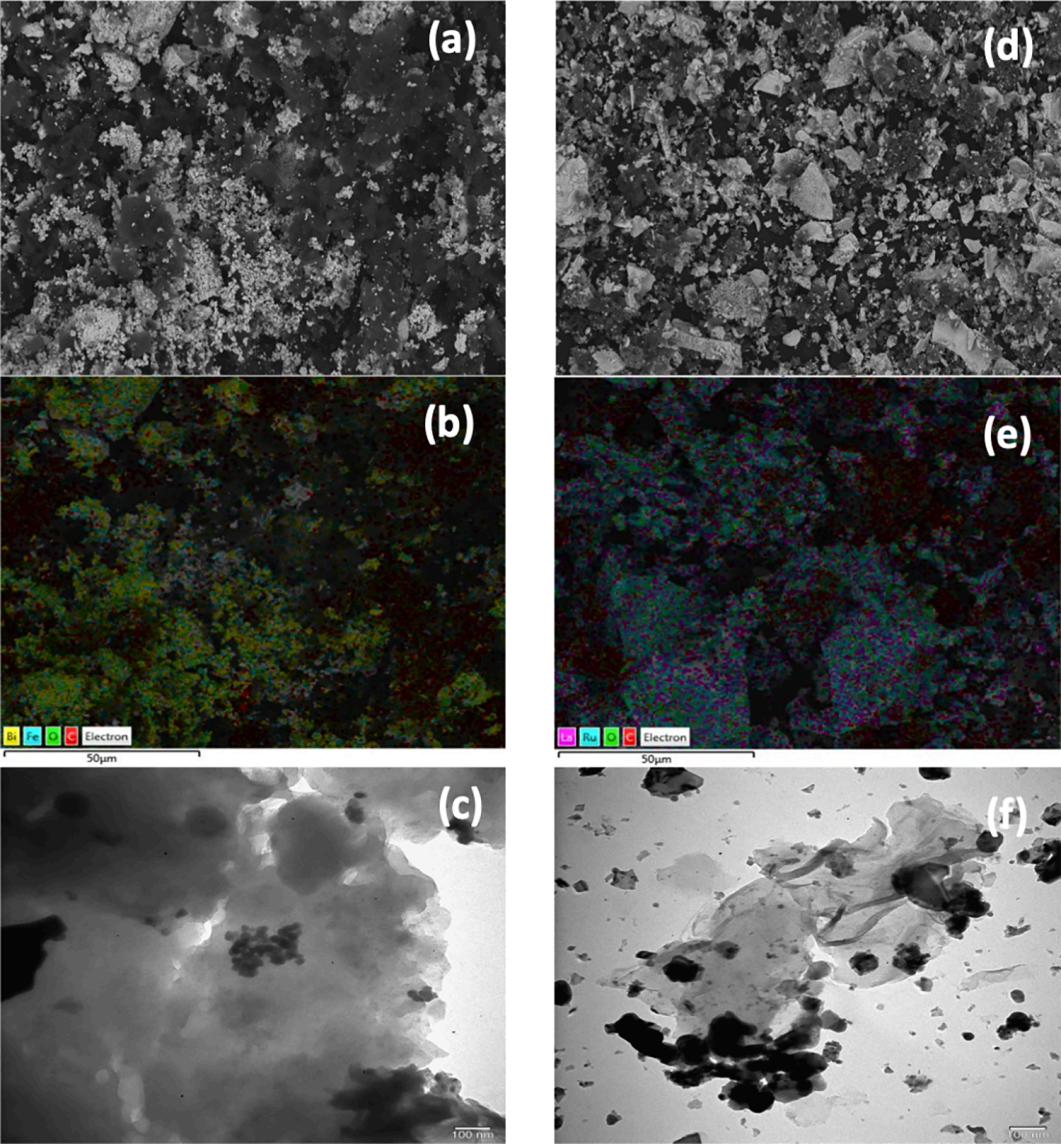
SEM images of GBFO (a) and GLRO (d); SEM-energy-dispersive spectrometry (EDS) spectra of GBFO (b) and GLRO (e); TEM images of GBFO (c) and GLRO (f).

**Figure 2:**
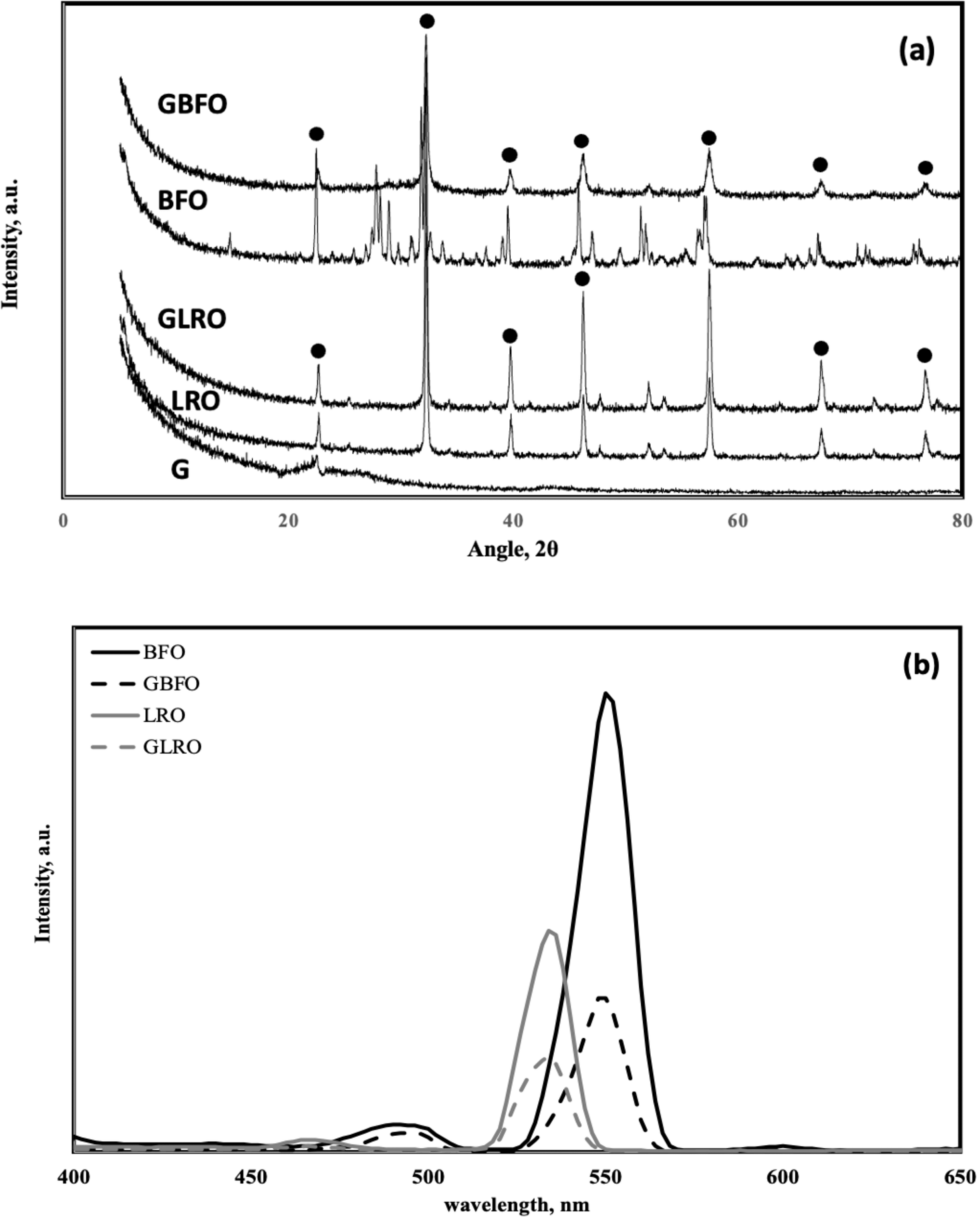
XRD results (a) and PL results (b).

The surface morphology of hybrid catalysts was investigated via scanning electron microscopy (SEM) analysis, and the results are shown in Figure 1. SEM images showed that they are agglomerated nanocrystals and have a porous structure. The perovskite structure did not change after the introduction of graphene to obtain hybrid catalysts. In addition, graphene was homogeneously distributed on the surface of hybrid catalysts, and in the literature, similar results were reported in many studies.^14,15^ Transmission electron microscopy (TEM) images of hybrid catalysts, shown in Figure 1, showed polycrystallinity and BFO and LRO covered in a layer of graphene, and the incorporation of graphene did not lead to any change in the structure of BFO and LRO. Brunauer–Emmett–Teller (BET) areas of perovskite and hybrid catalysts were determined. The BET area of BFO was 1.2 m^2^/g; however, it increased to 2.6 m^2^/g after the incorporation of graphene. On the other hand, LRO and GLRO had higher BET areas compared to BFO and GBFO. BET areas of LRO and GLRO were determined to be 22.7 and 27.6 m^2^/g, respectively. In the literature, the BET area of LRO was reported to be 2 m^2^/g by Pietri et al.;^16^ however, in the present study, it had a relatively higher BET area. Perovskite catalysts have characteristic peaks at certain 2θ values (22.63, 32.22, 39.73, 46.21, 57.45, 67.42, 72.12, and 76.69°), and the observation of these peaks at given 2θ values is attributed to the orthorhombic structure of perovskite-type catalysts.^14,15,17^ To confirm the presence of these characteristic peaks, X-ray diffraction (XRD) analysis was performed, and the results are shown in Figure 2. The results showed that all catalysts had characteristic peaks of perovskite structure, and similar results were reported in the literature.^18−20^ Additionally, the nanocrystalline nature of perovskite and hybrid catalysts was proved by the broadness of these peaks. Graphene has a characteristic peak around 21° and it has an amorphous structure,^21^ and the results showed that graphene was successfully synthesized. If the peak in the photoluminescence (PL) diagram of a photocatalyst has a reduced intensity, then it indicates a slower charge pair recombination and a longer life period of e^–^ and h^+^ pairs.^11,22−24^ Therefore, PL analysis was performed to comprehend the recombination process of photoexcited charge carriers and the life span of electron–hole pairs, and the results are shown in Figure 2. The peaks of BFO, GBFO, LRO, and GLRO were observed at 552, 550, 536, and 534 nm, respectively. Hence, the graphene incorporation led to a small shift and it could be concluded that lower photon energy is necessary to produce hydrogen using hybrid catalysts. Therefore, the highest hydrogen production should be observed in the presence of GLRO, while the lowest value should be observed using BFO.

**Figure 3:**
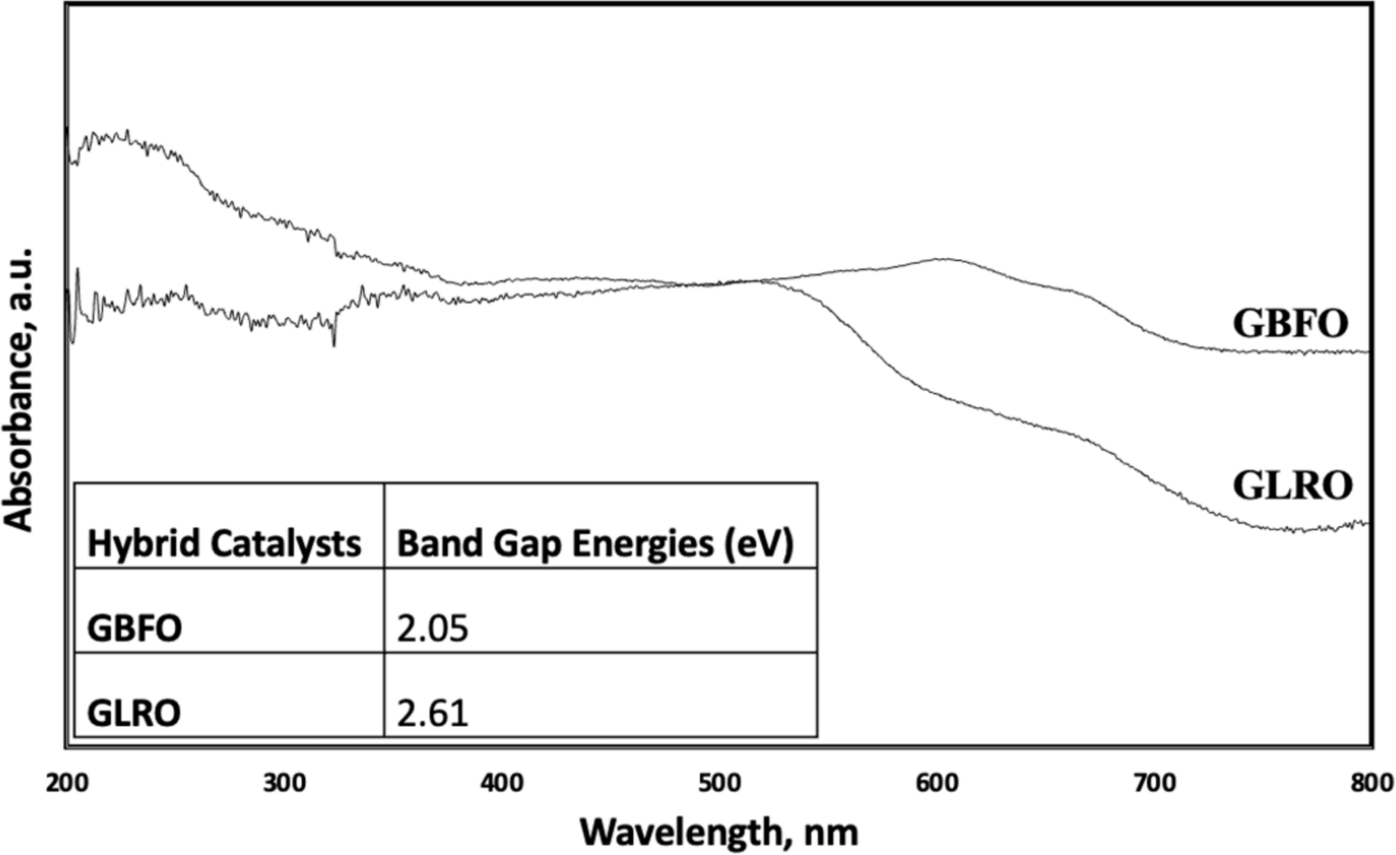
UV–vis diffuse reflectance spectra (DSR) results and band gap energies of hybrid catalysts.

UV–vis DRS analysis of GBFO and GLRO was carried out in the range of 200 and 800 nm, and their band gap energies were calculated by the Kubelka–Munk method. The results of this analysis are shown in Figure 3, and the band gap energies of GBFO and GLRO were determined to be 2.05 and 2.61 eV, respectively. In the literature, the band gap energy of BFO was reported to be between 2.2 and 2.8 eV. Additionally, the separation process of electron–hole pairs and the photocatalytic process for these hybrid catalysts and XPS results were reported in our previous study.^23^

**Figure 4:**
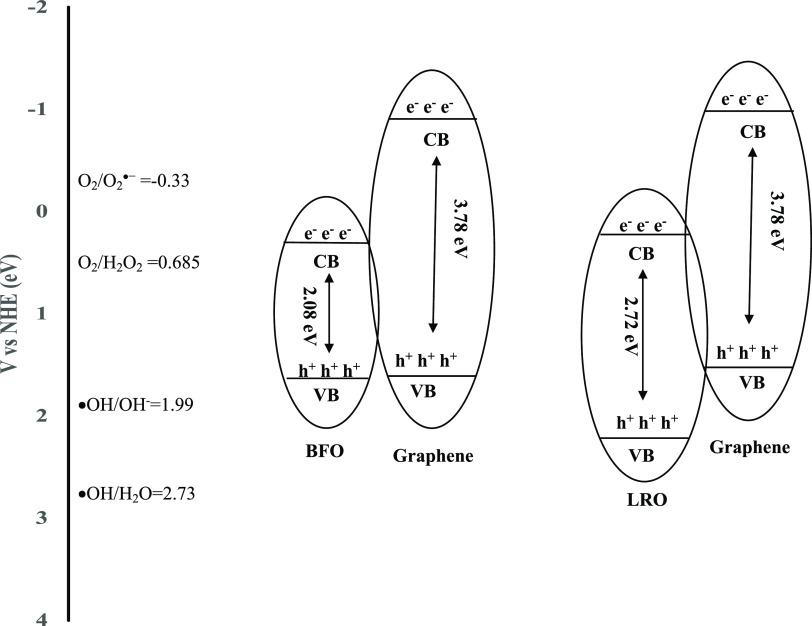
Schematic diagram of the separation process of electron–hole pairs of GBFO and GLRO.

In our previous study, the energy levels of calculated valence band edge (*E*_VB_) for BFO, LRO, and graphene were reported to be 2.58, 3.26, and 1.99, respectively. Additionally, the energy levels of calculated conduction band edge (*E*_CB_) for BFO, LRO, and graphene were reported to be 0.50, 0.45, and −1.79, respectively. Considering the results, it could be concluded that the CB position of BFO and LRO is lower than that of the graphene, which reduces the recombination of electron–hole pairs and enhances the photoactivity of GBFO and GLRO.^25^ A schematic diagram of the separation process of electron–hole pairs of GBFO and GLRO is shown in Figure 4. In this study, the introduction of graphene into the perovskite structure led to fast charge transfer, thereby enhancing the hydrogen evolution from sucrose model solution and sugar factory wastewater. Additionally, electrons are transferred through the graphene sheets to react with absorbed H^+^ into wastewater for hydrogen evolution. Various composite photocatalysts were used to produce hydrogen via photocatalysis. For instance, Chang et al. studied photocatalytic hydrogen evolution from lactic acid solution using MoS_2_/graphene under visible light. Similarly, they reported that the electrons can be transferred to the edge of MoS_2_ through the graphene sheets and then react with adsorbed H^+^ at the edges of MoS_2_ to form hydrogen.^26^

### Photocatalytic Hydrogen Evolution

Photocatalytic hydrogen evolution from sucrose solution and sugar industry wastewater was studied in the presence of GBFO and GLRO, and to examine the impact of reaction parameters (pH, catalyst amount (CA), and [H_2_O_2_]_0_) the Box–Behnken model was used. Additionally, the same experimental matrix was used for sucrose solution and sugar industry wastewater. The uncoded values of these parameters are 3–7.5, 0.1–0.2 g/L, and 0–15 mM for pH, catalyst amount, and initial hydrogen peroxide concentration, respectively. The uncoded design table of this experimental study and evolved hydrogen amounts (response) are presented in Table 1.

**Table 1:** Uncoded Design Table for Reaction Parameters and Response (Evolved Hydrogen)

			evolved hydrogen (μmol/g_cat_)			
reaction conditions			sucrose solution		sugar industry wastewater	
pH	catalyst amount [g/L]	initial H_2_O_2_ concentration [mM]	GBFO	GLRO	GBFO	GLRO
3	0.15	0	2717	3031	5490	5521
7.5	0.15	15	2580	3179	4472	4867
5.25	0.15	7.5	2701	3383	3564	3895
7.5	0.15	0	2756	2805	6089	6645
3	0.1	7.5	2552	2679	6617	6758
5.25	0.15	7.5	3102	3410	3245	3237
7.5	0.1	7.5	2569	2866	5429	5686
5.25	0.2	0	2591	3047	3674	3588
5.25	0.1	15	3515	3135	4032	4354
3	0.2	7.5	2729	3086	3339	3527
5.25	0.15	7.5	2739	3339	3823	4186
5.25	0.15	7.5	2673	3416	3124	3892
5.25	0.10	0	2970	2899	4296	4391
5.25	0.15	7.5	3460	3405	4208	4076
5.25	0.15	7.5	2662	3350	3075	4794
7.5	0.2	7.5	2783	2888	2778	2914
5.25	0.2	15	2508	3322	3174	3124
3	0.15	15	3025	3256	6436	6773

The evolved hydrogen amounts from sucrose solution in the presence of GBFO varied between 2778 and 6617 μmol/g_cat_, and the mean value was determined to be 2846 ± 21 μmol/g_cat_. The mean value of evolved hydrogen from sugar industry wastewater using GBFO was 3507 ± 469 μmol/g_cat_. The highest hydrogen production achieved was 6617 μmol/g_cat_ from sugar industry wastewater at pH 3 and using 0.1 g/L GBFO catalyst and 7.5 mM H_2_O_2_. This value was more than twice the amount of hydrogen obtained from the sucrose solution under the same reaction conditions. The mean value of evolved hydrogen from sucrose solution using GLRO was 3139 ± 34 μmol/g_cat_. The highest hydrogen evolution from sucrose solution was achieved at 3416 μmol/g_cat_ under the following reaction conditions: 5.25 pH, 0.15 g/L GLRO, and 7.5 mM [H_2_O_2_]_0_. The evolved hydrogen amounts from sugar industry wastewater using GLRO varied between 2914 and 6773 μmol/g_cat_. The mean value of evolved hydrogen from sugar industry wastewater using GLRO was 4013 ± 530 μmol/g_cat_. The highest hydrogen evolution obtained was 6773 μmol/g_cat_ from sugar industry wastewater at pH 3, 0.15 g/L GLRO, and 15 mM H_2_O_2_. This value was more than twice the amount of hydrogen obtained from sucrose solution under the same reaction conditions. Furthermore, the results were in consistence with the results of PL analysis of these catalysts.

The ANalysis Of Variance (ANOVA) table of this experimental design that was carried out using GBFO is presented in Table 2 to consider the interactions between factors based on the *p*-values of each factor. Based on the results, the most significant term was the [H_2_O_2_]_0_*[H_2_O_2_]_0_ and the main effects of reaction parameters (pH, catalyst amount, and [H_2_O_2_]_0_) were highly effective over the evolved hydrogen amount from sucrose solution using GBFO. Additionally, CA*[H_2_O_2_]_0_ was also efficient. The other terms did not have any impact on the evolved hydrogen statistically. The value of *R*^2^ for this model was calculated to be 98.82%, and hence it could be deduced that the model is a good fit to the observed response (evolved hydrogen amount). The main impact of pH and CA did not affect the evolved hydrogen amount from sugar industry wastewater; however, CA showed a main effect on the evolved hydrogen amount. Moreover, the square of pH was also statistically effective. Other parameters were not statistically effective on the evolved hydrogen amount from sugar industry wastewater using GBFO. In addition, the *p*-value (probability value) of lack-of-fit (0.079) was found to be higher than the value of α (0.05). Hence, it could be deduced that the model was well fitted to obtained experimental data. Furthermore, the *R*^2^ of the model was determined to be 86.35%, and thus it could be deduced that the model was a good fit to the observed response.

**Table 2:** ANOVA Table for Evolved Hydrogen Amounts Using GBFO

sucrose solution						sugar industry wastewater				
source	DF	Adj SS	Adj MS	*F*-value	*P*-value	DF	Adj SS	Adj MS	*F*-value	*P*-value
model	9	960 091	106 677	74.18	0.000001	9	22 089 149	2 454 350	5.62	0.012
linear	3	506 630	168 877	117.43	0.000001	3	8 331 329	2 777 110	6.36	0.016
pH	1	354 132	354 132	246.25	0.000000	1	1 211 588	1 211 588	2.78	0.134
CA	1	121 179	121 179	84.26	0.000016	1	6 862 106	6 862 106	15.73	0.004
[H_2_O_2_]_0_	1	31 319	31 319	21.78	0.001609	1	257 634	257 634	0.59	0.464
square	3	433 900	144 633	100.57	0.000001	3	12 002 961	4 000 987	9.17	0.006
pH*pH	1	74	74	0.05	0.825932	1	8 933 569	8 933 569	20.47	0.002
CA*CA	1	3301	3301	2.30	0.168244	1	686 803	686 803	1.57	0.245
[H_2_O_2_]_0_*[H_2_O_2_]_0_	1	414 035	414 035	287.90	0.000000	1	2 042 335	2 042 335	4.68	0.062
2-way Interaction	3	19 561	6520	4.53	0.038800	3	1 754 859	584 953	1.34	0.328
pH*CA	1	4728	4728	3.29	0.107381	1	98 302	98 302	0.23	0.648
pH*[H_2_O_2_]_0_	1	2731	2731	1.90	0.205529	1	1 642 571	1 642 571	3.76	0.088
CA*[H_2_O_2_]_0_	1	12 102	12 102	8.42	0.019863	1	13 986	13 986	0.03	0.862
error	8	11 505	1438			8	3 490 951	436 369		
lack-of-fit	3	9447	3149	7.65	0.025725	3	2 494 876	83 1625	4.17	0.079
pure error	5	2057	411			5	996 075	199 215		
total	17	971 596				17	25 580 099			

**Figure 5:**
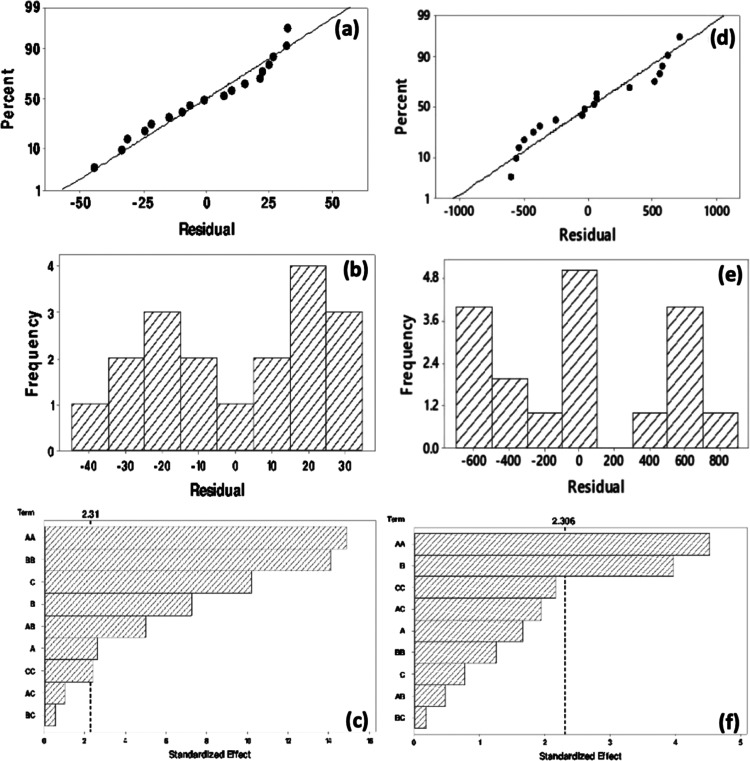
Normal probability plot (a), histogram (b), and Pareto chart (c) for evolved hydrogen amounts from sucrose solution using GBFO. Normal probability plot (d), histogram (e), and Pareto chart (f) for evolved hydrogen amounts from sugar industry wastewater using GBFO.

The normal probability plot and histogram diagram are shown in Figure 5a and b, and the Pareto chart is shown to comprehend the individual and interaction effects of factors in Figure 5c for evolved hydrogen amount from sucrose solution using GBFO. In addition, the probability plot, histogram diagram, and Pareto chart for evolved hydrogen amount from sucrose solution using GBFO are shown Figure 5d–f, respectively.

Experimental points were reasonably aligned, suggesting normal distribution between −60 and +60 in Figure 5a. In Figure 5b, the *t*-value was 2.31 in a confidence level of 95%. Hence, [H_2_O_2_]_0_*[H_2_O_2_]_0_ was the most important parameter. In addition, all main factors also had a major effect on the photocatalytic hydrogen evolution from sucrose solution using GBFO. Moreover, CA*[H_2_O_2_]_0_ had a synergistic effect on the evolved hydrogen amount from sucrose solution using GBFO. The normal probability plot (Figure 5d) implies that the data were normally distributed. Additionally, Figure 5e shows that the experimental points were aligned in the range of −600 and +800. The normal distribution range extended in a larger interval compared to the results of sucrose solution. The main and interaction effects of the reaction parameter were elucidated via Pareto chart, and the minimum statistically important impact magnitude for 95% confidence level was shown with the vertical line in Figure 5f. Thus, the parameter which has a higher magnitude than this vertical line was statistically effective over hydrogen evolution from sugar industry wastewater using GBFO. In addition, the *t*-value is equal to 2.306 in this confidence level. Therefore, among the main effects, the only effective parameter was CA for the photocatalytic hydrogen evolution from sugar industry wastewater using GBFO. In addition, the squares of pH affected the evolved hydrogen amount. However, other parameters were not statistically effective.

The ANOVA table of this experimental design that was carried out using GLRO is presented in Table 3 to comprehend the main effects of factors and the interactions between them based on *p*-values of each factor. The main effects and the square of all factors were significant over the evolved hydrogen amount from sucrose solution using GLRO. Among two-way interaction terms, pH*CA had a synergistic effect and the other terms did not affect the amount of evolved hydrogen from sucrose solution statistically. The main effect of pH and [H_2_O_2_]_0_ did not affect the evolved hydrogen amount; however, CA showed a main effect. Moreover, the square of pH was also statistically effective. Among the two-way interactions, pH*[H_2_O_2_]_0_ had a significant effect over the evolved hydrogen amount from sugar industry wastewater using GLRO. Therefore, the pH and [H_2_O_2_]_0_ showed a synergetic effect during the degradation of organic compounds in the sugar industry wastewater and concomitant hydrogen evolution. The synergetic effect of pH and [H_2_O_2_]_0_ depends on various parameters such as TOC and concentration of organic substances in the wastewater stream, and if their synergetic effect is observed, it implies that appropriate reaction conditions were provided. Therefore, the reaction conditions were appropriate to produce hydrogen and degrade organic substances in the wastewater. However, other parameters did not have a statistically significant effect over the evolved hydrogen amount using GLRO. The *p*-value of lack-of-fit (0.155) is higher than the value of α (0.05), and thus it could be inferred that the model was well fitted to the experimental data. In addition, the *R*^2^ values for this model were determined to be 98.84 and 87.26% for sucrose solution and sugar industry wastewater, respectively. Therefore, the model showed a good fit to the observed response data.

**Table 3:** ANOVA Table for Evolved Hydrogen Amounts Using GLRO

sucrose solution						sugar industry wastewater				
source	DF	Adj SS	Adj MS	*F*-value	*P*-value	DF	Adj SS	Adj MS	*F*-value	*P*-value
model	9	874 526	97 170	75.60	0.00000	9	22 935 030	2 548 337	6.09	0.009
linear	3	210 295	70 098	54.54	0.00001	3	8 964 259	2 988 086	7.14	0.012
pH	1	8714	8714	6.78	0.03143	1	760 841	760 841	1.82	0.214
CA	1	67 910	67 910	52.84	0.00009	1	8 071 258	8 071 258	19.29	0.002
[H_2_O_2_]_0_	1	133 671	133 671	104.00	0.00001	1	132 160	132 160	0.32	0.590
square	3	630 624	210 208	163.55	0.00000	3	11 577 791	3 859 264	9.22	0.006
pH*pH	1	284 963	284 963	221.72	0.00000	1	8 523 058	8 523 058	20.37	0.002
CA*CA	1	255 119	255 119	198.50	0.00000	1	2 076 506	2 076 506	4.96	0.057
[H_2_O_2_]_0_*[H_2_O_2_]_0_	1	7344	7344	5.71	0.04383	1	1 275 205	1 275 205	3.05	0.119
2-way interaction	3	33 607	11 202	8.72	0.00668	3	2 392 981	797 660	1.91	0.207
pH*CA	1	31 958	31 958	24.87	0.00107	1	52 829	52 829	0.13	0.732
pH*[H_2_O_2_]_0_	1	1278	1278	0.99	0.34782	1	2 294 740	2 294 740	5.48	0.047
CA*[H_2_O_2_]_0_	1	371	371	0.29	0.60587	1	45 412	45 412	0.11	0.750
error	8	10 282	1285			8	3 347 946	418 493		
lack-of-fit	3	4932	1644	1.54	0.31423	3	2 073 184	691 061	2.71	0.155
pure error	5	5350	1070			5	1 274 762	254 952		
total	17	884 808				17	26 282 976			

**Figure 6:**
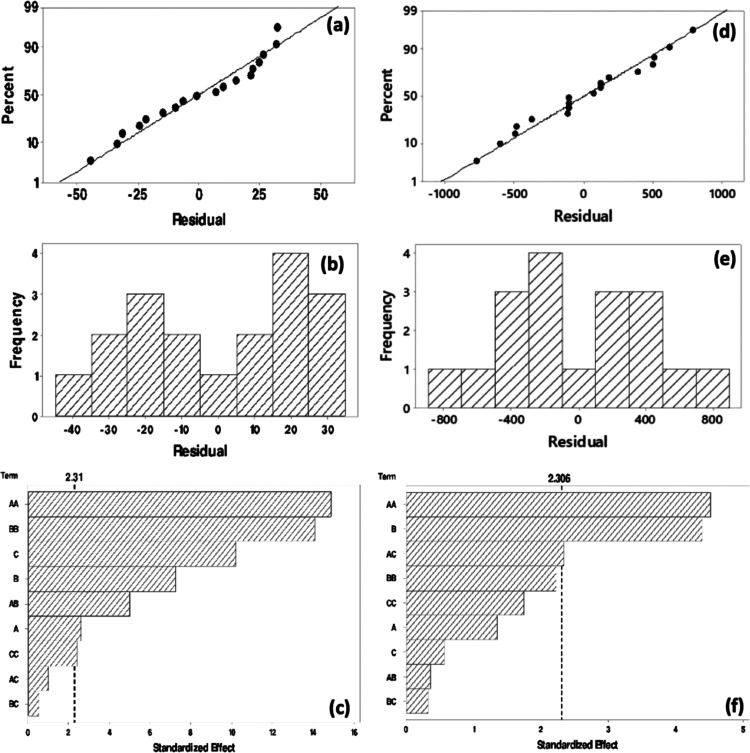
Normal probability plot (a), histogram (b), and Pareto chart (c) for the evolved hydrogen amount from sucrose solution using GLRO. Normal probability plot (d), histogram (e), and Pareto chart (f) for the evolved hydrogen amount from sugar industry wastewater using GLRO.

Based on the residual plots, it could be deduced that the experimental points for evolved hydrogen from sucrose solution (Figure 6a) and from sugar industry wastewater (Figure 6d) were reasonably aligned with normal distribution between −40 and +30 and in the range of −800 and +800, respectively. Pareto charts of evolved hydrogen from sucrose solution and from sugar industry wastewater are shown in Figure 6c and f, respectively. In these graphs, the *t*-value is equal to 2.31 with a confidence level of 95%. Therefore, the square of pH and the square of CA were the most important parameters for the photocatalytic hydrogen evolution from sucrose solution using GLRO. Additionally, the square of [H_2_O_2_]_0_ was also an effective parameter. Moreover, all main factors also had a major effect over the evolved hydrogen amount. Moreover, the two-way interaction of pH and CA had a synergistic effect over the produced hydrogen amount. On the other hand, among the main effects, the only effective parameter was CA for the photocatalytic hydrogen evolution from sugar industry wastewater using GLRO. Although the squares of pH and the two-way interaction of pH and [H_2_O_2_]_0_ affected the evolved hydrogen amount, other factors were not statistically effective.

## Conclusions

Photocatalytic oxidation is a feasible process to produce hydrogen, which is a clean and green fuel from renewable sources. In this context, first, solar-driven hybrid catalysts (graphene-supported BiFeO_3_ and graphene-supported LaRuO_3_) were synthesized. Then, the characterization study consisting of SEM, TEM, BET, XRD, PL, and UV–vis DSR analyses was conducted, and based on PL results, GLRO was the most promising photocatalyst to produce hydrogen. The Box–Behnken experimental design model was used to investigate the main and interaction effects of reaction parameters (pH, catalyst loading, and [H_2_O_2_]_0_) on the evolved hydrogen amount from sucrose solution and sugar industry wastewater via photocatalysis. The lowest evolved hydrogen amount was 2508 μmol/g_cat_ at the following reaction conditions: a pH of 5.25, 0.2 g/L catalyst (GBFO) loading, and 15 mM [H_2_O_2_]_0_, while the highest hydrogen evolution achieved was 6773 μmol/g_cat_ from sugar industry wastewater at pH 3, 0.15 g/L catalyst (GLRO) loading, and 15 mM [H_2_O_2_]_0_. The most effective parameter over evolved hydrogen amount using GLRO was found to be the square of pH, and the other effective parameters were catalyst amount and two-way interaction of pH and [H_2_O_2_]_0_. It could be deduced that pH and [H_2_O_2_]_0_ showed a synergetic effect over the evolved hydrogen amount from sugar industry wastewater using GLRO.

## Materials and Methods

### Materials

Sucrose (Merck) and sugar industry wastewater (supplied from Eskişehir Sugar Factory, Turkey) were used to produce hydrogen. Graphite, sodium nitrate, hydrogen peroxide, lanthanum nitrate hexahydrate, bismuth nitrate pentahydrate, iron nitrate nonahydrate, hydrazine hydrate, and sodium hydroxide were purchased from Merck to conduct the study. Citric acid and ethylene glycol were purchased from Isolab, and potassium permanganate was purchased from Tekkim. Lastly, ruthenium chloride and hydrochloric acid were purchased from Sigma.

### Synthesis of Hybrid Catalysts

First, graphene was produced from graphite via the chemical decomposition method. In this context, 1 g of graphite was mixed with NaNO_3_ (0.5 g) and sulfuric acid (25 mL) and then the mixture was kept in an ice bath to control the temperature (*T* < 20 °C) during the addition of KMnO_4_ (3 g) into this mixture. After that, this solution was still kept in the ice bath and stirred at 35 °C for 40 min. After completing this step, 45 mL of distilled water (DW) was put in a beaker and the obtained solution was added into DW and stirred for 20 min. Then, a mixture of H_2_O_2_ and DW (140:10, v/v) was prepared and preprepared graphite-containing solution was added into this H_2_O_2_ and DW solution. The final solution was kept at room temperature for 1 day and then it was filtered, and hence graphene oxide (GO) was obtained. Then, to remove the impurities (i.e., metal ions, sulfate, etc.), it was washed with 5% HCl solution and the solid residue was calcinated at 300 °C for 15 min. After this step, hydrazine hydrate (HH) was used to reduce GO to graphene; 1 mL of HH was added to 100 mg of GO (on basis), and the mixture of HH and GO was stirred at 80 °C for 6 h. Then, the obtained solid residue was dried under vacuum at 50 °C to afford graphene.^27^

Second, perovskite-type catalysts (BiFeO_3_ and LaRuO_3_) were synthesized and detailed synthesis steps were given. To prepare BiFeO_3_, bismuth nitrate and iron nitrate were used as precursors and they were separately dissolved in DW considering their stoichiometric ratio. After that, they were mixed together and stirred to obtain a precursor solution at room temperature for 1 h. A mixture of citric acid (1.5 times of total moles of precursors) and ethylene glycol (25 mL for 0.1 mol of each precursor) in DW was prepared and added into the obtained precursor solution. Then, it was heated up to 80 °C by stirring, and it was kept at 80 °C until the gel formation was observed. Thereafter, the gel was dried at 150 °C for 6 h, and then it was calcinated at 700 °C for 4 h to obtain BiFeO_3_ (BFO).^28^ To synthesize LaRuO_3_ (LRO), lanthanum nitrate and ruthenium chloride were used. First, citric acid, ruthenium chloride, and ethylene glycol were mixed at the given ratios (citric acid/ruthenium chloride = 4 and ethylene glycol/citric acid = 1.38). Then, lanthanum nitrate was added considering the stoichiometric ratio between lanthanum and ruthenium into this solution and kept at 50 °C and complete evaporation was achieved after 2 days. Then, the remaining residue was dried at 150 °C for 6 h, and then it was calcinated at 700 °C for 4 h to obtain LaRuO_3_ (LRO).^16^

Last, hybrid catalysts (GBFO and GLRO) were synthesized using previously prepared graphene and perovskite catalysts. To prepare 20 wt % graphene-containing hybrid catalysts, first, the required amount of graphene was added into the ethanol/water mixture and then sonicated for 2 h. After sonication, the perovskite catalyst (i.e., BFO) was added into this solution and stirred for 2 h. Then, the solid residue was separated via centrifugation and dried at 70 °C for 12 h.^28,29^ Consequently, the hybrid catalysts were obtained.

### Characterization of Hybrid Catalysts

SEM-EDS analysis with the FEI QUANTA 250 FEG model was performed to investigate the surface morphology of hybrid catalysts. Also, TEM (JEOL 2100F 200 kV RTEM) analysis was performed. The BET areas of hybrid catalysts were determined using a Micromeritics ASAP 2010. The crystalline structure of hybrid catalysts was analyzed via XRD (Philips X’Pert diffractometer with Cu Kα radiation, 2θ = 5–80°, step length: 0.02°). PL (Edinburgh Instruments FLSP920) was carried out to determine the photocatalytic activities of hybrid catalysts. UV–vis DSR (UV–vis, Shimadzu UV 2600) was used to calculate the band gap energies of hybrid catalysts.

### Experimental Setup and Procedure

Photocatalytic hydrogen production setup consists of a cylindrical glass reactor (*V* = 1000 mL) with a jacket, a pump for circulation of solution, a stimulated solar lamp placed in the middle of a glass reactor, and a gas analysis system (ABB Advance Optima, AO2000) connected to the gas outlet of the reactor to measure the amounts of produced gases (CO, CO_2_, H_2_, and CH_4_) throughout the experiments. A typical experimental procedure started with introducing the sucrose solution or sugar industry wastewater at desired pH value (adjusted using HCl or NaOH solution) in the reactor. After that, the desired amount of hybrid catalyst and hydrogen peroxide were introduced, and then the stimulated solar lamp was turned on to start the reaction to produce hydrogen via photocatalysis. The amounts of produced gases throughout the reaction were measured with a gas analysis system and the experiments lasted up to 4 h.
